# Are rural pregnant women disadvantaged in accessing intermittent preventive treatment in pregnancy in Ebonyi State, Nigeria?

**DOI:** 10.1371/journal.pone.0269305

**Published:** 2022-11-10

**Authors:** Christian Obasi Akpa, Benedict Ndubueze Azuogu, Winifred Chinwendu Akpa, Chukwuma David Umeokonkwo, Chiaka Patience Denwigwe, Victoria Chioma Azuogu, Adaoha Pearl Agu, Onwe Emeka Ogah, Ifeyinwa Chizoba Akamike, Ituma Bernard Ituma, Adanna Anthonia Umeokonkwo, Cosmas Kenan Onah, Azuka Stephen Adeke, Ifeoma Sophia Usuwa, Lawrence Ulu Ogbonnaya, Edmund Ndudi Ossai, Chihurumnanya Alo, Chika Onwasigwe, Benjamin Sunday Uzochukwu

**Affiliations:** 1 Department of Community Medicine, Alex Ekwueme Federal University Teaching Hospital, Abakaliki, Ebonyi State, Nigeria; 2 Department of Medical Microbiology, Ebonyi State University, Abakaliki, Ebonyi State, Nigeria; 3 Department of Guidance and Counseling, University of Calabar, Calabar, Cross River State, Nigeria; 4 Department of Nursing Education, Ebonyi State University, Abakaliki, Ebonyi State, Nigeria; 5 Department of Paediatrics, Alex Ekwueme Federal University Teaching Hospital, Abakaliki, Ebonyi State, Nigeria; 6 Department of Family Medicine, University of Uyo Teaching Hospital, Uyo, Akwa Ibom State, Nigeria; 7 Department of Community Medicine, University of Nigeria Teaching Hospital, Enugu, Nigeria; University of Washington, UNITED STATES

## Abstract

**Introduction:**

Adequate intermittent preventive treatment (IPTp) uptake (≥3 doses) routinely delivered at antenatal clinics is effective in preventing malaria during pregnancy. Whereas, low IPTp uptake (24.0%) had been reported among pregnant women in Ebonyi State, there is paucity of studies comparing the uptake and its predictors in the urban and rural areas of Ebonyi State. We determined IPTp uptake and its predictors in the urban and rural areas of Ebonyi State.

**Methods:**

We conducted a cross-sectional comparative study among 864 reproductive age women selected using multistage sampling. Using a structured interviewer-administered questionnaire, we collected data on respondent’s socio-demographic characteristics and IPTp uptake. Uptake was adjudged adequate if ≥3 doses were taken, otherwise inadequate. We estimated the proportion of women with adequate IPTp uptake and determined the factors associated with adequate uptake in rural and urban areas using chi square and multiple logistic regression at 5% level of significance.

**Results:**

The mean ages of respondents in the urban and rural areas were 28.5±4.6 and 27.4±5.0 years respectively. Adequate IPTp uptake was 82.5% and 60.8% in the urban and rural respectively (p<0.001). In the urban area, women whose husbands had attained ≥ secondary education (aOR:2.9; 95%CI:1.2–7.4; p = 0.02) and those who paid for sulfadoxine/pyrimethamime (aOR:0.2; 95%CI: 0.1–0.6; p = 0.01) were 2.9 times more likely and 5 times less likely to take adequate IPTp respectively compared to respondents whose husbands had attained ≤ primary education and those who had sulfadoxine/pyrimethamine free. In the rural area, women who had attended ANC <4 times (aOR:0.4; 95%CI: 0.3–0.7; p<0.001) were 2.5 times less likely to take adequate IPTp compared to women that had attended ANC ≥4 times.

**Conclusion:**

Uptake of IPTp was more in the urban than rural areas of Ebonyi State. Interventions that reinforce the importance of health professionals carrying out actions aimed at pregnant women and their partners (spousal) in order to guide them on preventive actions against malaria and other diseases are recommended in Ebonyi State.

## Background

The World Health Organization recommends intermittent preventive treatment of malaria using Sulfadoxine-Pyrimethamine (SP) in places of stable malaria transmission in Africa as one of the ways to prevent morbidity and mortality arising from malaria in pregnancy [1]. It is estimated that each year approximately 25 million pregnant women in sub-Saharan Africa live at risk of P. falciparum malaria infection [2]. Malaria infection in pregnancy compromises the mother’s health and can lead to her death. In 2018, an estimated 11 million pregnant women living in 38 countries with moderate-to-high transmission in sub-Saharan Africa were infected with malaria (29% of all pregnancies) [3].

Adequate uptake (≥3 doses) of intermittent preventive treatment (IPTp) has been shown to be effective in prevention of malaria during pregnancy [1]. IPTp is taken one month apart, starting at 16 or 18 weeks of gestation and routinely delivered at antenatal clinics. Low uptake had been reported been reported in sub-Saharan Africa including Ghana in 2019 [4, 5]. Nigeria embraced IPTp strategy in 2005 [6] but the coverage is still below optimal and the level of uptake differs in the urban and rural areas across some States in Nigeria. In 2018 National Demographic and Health Survey, the proportion of pregnant women who took at least one dose of SP was 63.6%, (72.6% urban and rural 58.0%) more so, the proportion of pregnant women who took at least three doses of SP was revealed as 16.6% (urban 20.7% and rural 14.0%) [7]. However, the predictors of adequate uptake of IPTp were not reported. A study in Enugu, Nigeria in 2012 on IPTp uptake among 1307 study participants revealed that uptake remained low though generally but lower in the rural than urban area [8]. In 2018, It was also reported that 24.2% received at least three doses of SP in Ebonyi State [9]. Whereas, low IPTp uptake had been reported among pregnant women in selected communities of Ebonyi State, there appears to be paucity of studies documenting and comparing its predictors in the urban and rural areas of Ebonyi State [10]. This study determined and compared the level of IPTp uptake and its predictors in the urban and rural areas of Ebonyi State, Nigeria.

## Methods

### Study area

The study was conducted in Ebonyi State. The State capital is Abakaliki. Other major towns include Afikpo, Okposi and Onueke. There are 3 senatorial zones (South, North and Central) and 13 Local Government Areas in the State with the 2019 projected population of Ebonyi State and population of women of reproductive age were 3,112,220 and 684,688 respectively as obtained from the State Ministry of Health [11, 12].

There are only four urban Local Government Areas (LGAs) in Ebonyi State out of the 13 LGAs that make up the state. The urban LGAs are Abakaliki, Afikpo North, Ebonyi and Ezza South [13]. The remaining nine are rural LGAs. In this context, an urban area is a continuously built-up area with a population of 50,000 or more. It comprises one or more places (central place(s)) and adjacent densely settled surrounding area (urban fringe) consisting of other places and non-place territory [14]. A rural area is any place, and housing units that is not classified as an urban area [14].

There are 556 public and private health facilities in Ebonyi State, comprising of 13 general hospitals (4 in the urban LGAs and 9 in the rural), 6 mission hospitals (2 in the urban LGAs and 4 in the rural), 417 primary health centres (128 in the urban LGAs and 289 in the rural) and 119 private hospitals/clinics mostly distributed in the urban LGAs [11]. Most of these health facilities offer maternal and child health services such as antenatal care (ANC) and immunization services. In Ebonyi State, at 2018, the ANC attendance at health facilities with skilled providers was 70.3% [9]. Average monthly attendance of women bringing their children for immunization in the selected Health facilities in the past 6 months rural Health facilities is 1223 and 1010 for urban Health facilities in Ebonyi State [13]. Malaria control in Ebonyi State are currently being supported by Roll Back Malaria Programme and President’s Malaria Initiative. The State Ministry of Health with the support of the Federal Ministry of Health supplies Sufadixine-Pyrimethamine and insecticide treated nets (ITN) to the Health facilities in Ebonyi State through pull method. There exists a policy in Ebonyi State that these commodities supplied by the State should be given free to pregnant women during ANC [11].

### Study design and population

We conducted a cross-sectional comparative study among women of reproductive age. We included women who had been living in the selected areas, attended ANC, gave birth at the selected Health facilities in the past year prior to the survey. Women who were not disposed to respond to the interview; those who were on cotrimoxazole (septrin) tablet during pregnancy and those less than 18 years were excluded.

### Sample size determination

The minimum sample size for each group was calculated using the formula below [15]:

$$\text{n}=\frac{{\left[\text{Z}\alpha +\text{Z}\beta \right]}^{2}\;\text{x}\;\left[{\text{P}}_{1}\left(1-{\text{P}}_{1}\right)+{\text{P}}_{2}\left(1-{\text{P}}_{2}\right)\right]}{{\left[{\text{P}}_{1}-{\text{P}}_{2}\right]}^{2}}$$

Where:

n = minimum sample size in each group

Zα = 1.96, the critical ratio or Standard normal deviation at significance level of 5%

Zβ = 0.84, the critical ratio or standard normal deviate at desired power of 80%

P_1_ = proportion of coverage of IPTp in urban areas in Nigeria Malaria

Indicator Survey, P_1_ = 24.0% [16].

P_2_ = proportion of coverage of IPTp in rural area in Nigeria Malaria

Indicator Survey, P_2_ = 16.0% [16].

A sample of 432 participants was estimated for each group after adjusting for 10% non-response rate.

### Sampling technique

We used multistage sampling to recruit participants for the study. The 13 LGAs were first stratified into four urban and nine rural LGAs. Two LGAs each were selected by balloting from each stratum, two urban (Abakaliki and Ebonyi) and two rural (Izzi and Ohaozara). In each of the 2 urban and 2 rural LGAs selected in stage 1, a list of the health facilities that fall into them from the 556 Health facilities in Ebonyi State was made and this served as the sampling frame. Four health facilities were selected in each of the LGAs by balloting method of simple random sampling giving a total of 16 Health facilities. These include Primary Health Centre (PHC) Azuiyiokwu, PHC Izzi Unuhu, PHC Onuebonyi and PHC Unagboke for Abakaliki LGA; Alex Ekwueme Federal University Teaching Hospital, Abakaliki (AEFUTHA), Mile 4 hospital, PHC Nsugbe and Chidera hospital Kpirikpiri for Ebonyi LGA; St Vincent hospital, PHC Iboko, PHC Nwezenyi and PHC Ndiechi for Izzi LGA and PHC Obiozara, PHC Egugwu, PHC Onuaviankwo and PHC Okposi Court Area for Ohaozara LGA. Proportionate allocation with attendance for immunization at the selected Health facilities was done to determine the number of respondents that were selected per Health facility. In each of the health facilities selected in stage 2, the women attending immunization in each of the selected Health facilities were listed each day and this formed the sampling frame, respondents were selected randomly (through balloting) from them till the number of respondents allotted to each was met. However, in Health facilities where the number of eligible women was not met, a 2^nd^ visit was made, and the process repeated to get the number of eligible women proportionately allocated to them.

### Measurement of variables

The dependent variable was IPTp uptake. This was categorized based on the number of doses of IPTp they took during the pregnancy in the year preceding the study. Those who took less than three doses (<3 doses) were categorized as inadequate and those who took three doses and above (≥3 doses) throughout the duration of their pregnancy were categorized as having had adequate uptake. To examine and compare the predictors of IPTp uptake in the urban and rural areas, the categories remained inadequate and adequate uptake. The independent variables were age, education level, Trimester at 1^st^ ANC attendance, number of ANC attendance and payment for IPTp.

### Study tool

We developed the questionnaire for this study. Some part of the structured questionnaire was adapted from the women questionnaire part of the 2018 Nigeria Demographic Health Survey (NDHS) [7]. The questionnaire has different sections which assessed the socio-demographic characteristics of participants, uptake of intermittent preventive treatment for malaria in pregnancy, mode of IPTp uptake, health facility type and spousal information in the urban and rural areas. The questionnaire is not under any copyright protection. It was pretested among 50 women in a health facility not selected for the study. From the result of the pretesting, some questions were modified for clarity. The NDHS survey tool has been previously validated in Nigeria; the authors did not conduct any further validation on the tool. A copy of the questionnaire is included in the manuscript.

### Data collection and analysis

The questionnaire was interviewer administered by trained research assistants for one month. It was administered as exit interview during the children immunization visits. Data were analyzed with Statistical Package for Social Science (IBM SPSS) version 20. We estimated the proportion of the women who received IPTp during their last pregnancy in urban and rural areas. We also estimated the proportion of women that received adequate IPTp in urban and rural areas. We examined the association between adequate IPTp uptake and socio-demographic characteristics using Chi Squared test in the urban and rural areas. The variables that had a p value of ≤0.1 in the bivariate analysis were included in the multiple logistic regressions. The predictors of adequate IPTp uptake were identified in the urban and rural areas at 5% level of significance.

### Ethical considerations

Ethical approval was obtained from the Research and Ethics Committee of Federal Teaching Hospital Abakaliki, Ebonyi State (approval reference number: FETHA/REC/VOL 2/2019/176). We obtained written informed consent from all participants after explaining the details of the study. Participation in the study was voluntary and strict confidentiality was ensured in handling the information obtained in the study. At the end of data collection health education sessions were carried out in all the health facilities used for the study. Recommendations from the findings in this study were made available to the Ministry of Health of Ebonyi State to help in influencing policy making and strengthening of IPTp uptake in Ebonyi State.

## Results

The mean ages of respondents in the urban and rural areas were 28.5±4.6 and 27.4±5.0 years respectively. Majority of the respondents were within the age group 25–34 years in the urban (67.8%) and rural (59.5%) areas. Most of the participants were married in both urban (99.5%) and rural (94.9%) areas. Majority of the respondents had attained secondary education and above in the urban area (68.3%) while most had attained primary education and less (65.7%) in the rural area. Majority of the respondents’ husbands had attained secondary education and above in the urban area (91.8%) and (90.1%) in the rural area (Table 1).

**Table 1:** Socio-demographic and reproductive characteristics of respondents.

Variables	Urban (n = 432) (%)	Rural (n = 432) (%)
**Age (years)**		
18–24	88 (20.4)	126 (29.2)
25–34	293 (67.8)	257 (59.5)
≥35	51 (11.8)	49 (11.3)
**Mean age**	28.5±4.6	27.4±5.0
**Marital status**		
Married	430 (99.5)	410 (94.9)
Unmarried	2 (0.5)	22 (5.1)
**Respondent education**		
None	20 (4.5)	55 (12.7)
Primary	118 (27.2)	230 (53.0)
Secondary	157 (36.3)	124 (29.0)
Tertiary	137 (32.0)	23 (5.3)
**Husband education**		
No formal education	12 (2.8)	7 (1.7)
Primary	23 (5.4)	34 (8.2)
Secondary	108 (25.1)	203 (49.3)
Tertiary	287 (66.7)	168 (40.8)
**Respondent occupation**		
Trader	160 (37.0)	94 (46.5)
Civil servant	119 (27.6)	47 (10.9)
Artisan	99(22.9)	94 (21.8)
Student	22 (5.1)	13 (3.0)
No occupation	20 (4.6)	39 (9.0)
Farmer	11 (2.6)	37 (8.6)
Force	1 (0.2)	1(0.2)
**Husband occupation**		
Civil Servant	202 (47.0)	91 (22.0)
Artisan	116 (27.0)	149 (36.2)
Trader	99 (23.0)	115 (27.9)
Farmer	7 (1.6)	53 (12.9)
Force	4 (0.9)	4 (1.0)
Student	2 (0.5)	0 (0.0)
**Husband’s employment**		
Employed	243 (56.5)	303 (73.5)
Unemployed	187 (43.5)	109 (26.5)
**Family type**		
Monogamy	413 (96.0)	370 (89.8)
Polygamy	17 (4.0)	42 (10.2)
**Trimester at 1**^**st**^ **ANC attendance of the last pregnancy**		
1^st^ trimester	163 (37.7)	158 (36.5)
2^nd^ trimester	244(56.5)	240 (55.5)
3^rd^ trimester	25 (5.8)	34 (8.0)
**Number of ANC attended during the last pregnancy**		
≥ 5 times	250 (57.9)	203 (47.0)
4 times	94 (21.8)	89 (21.0)
3 times	74 (17.1)	84 (19.0)
2 times	13 (3.0)	42 (10.0)
Once	1 (0.2)	14 (3.0)

The predominant family type was monogamy in both the urban area (96.0%) and (89.8%) in the rural area, Table 1. Most of the respondents attended ANC ≥5 times in the urban area (57.9%) and (47.0%) in the rural area, Table 1.

**Fig 1:**
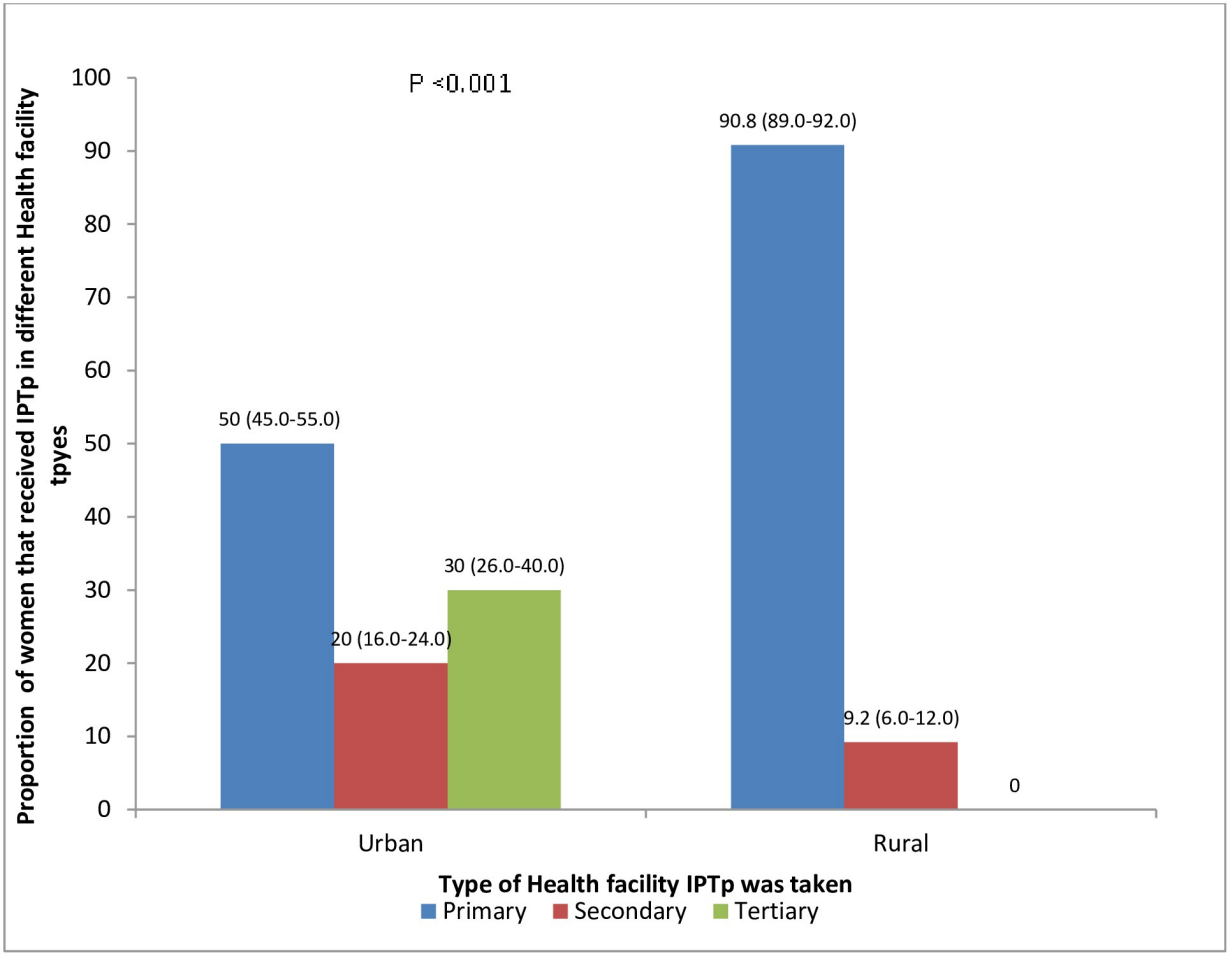


Distribution of participants according to the type of Health facility where intermittent preventive treatment of malaria in pregnancy (IPTp) was received in the urban and rural areas shows that about half of the respondents took IPTp in the primary health facility in the urban while majority in the rural area (90.8%) had it in the primary health facilities (x^**2**^ = 137.480; p-value = <0.001) (Fig 1).

**Fig 2:**
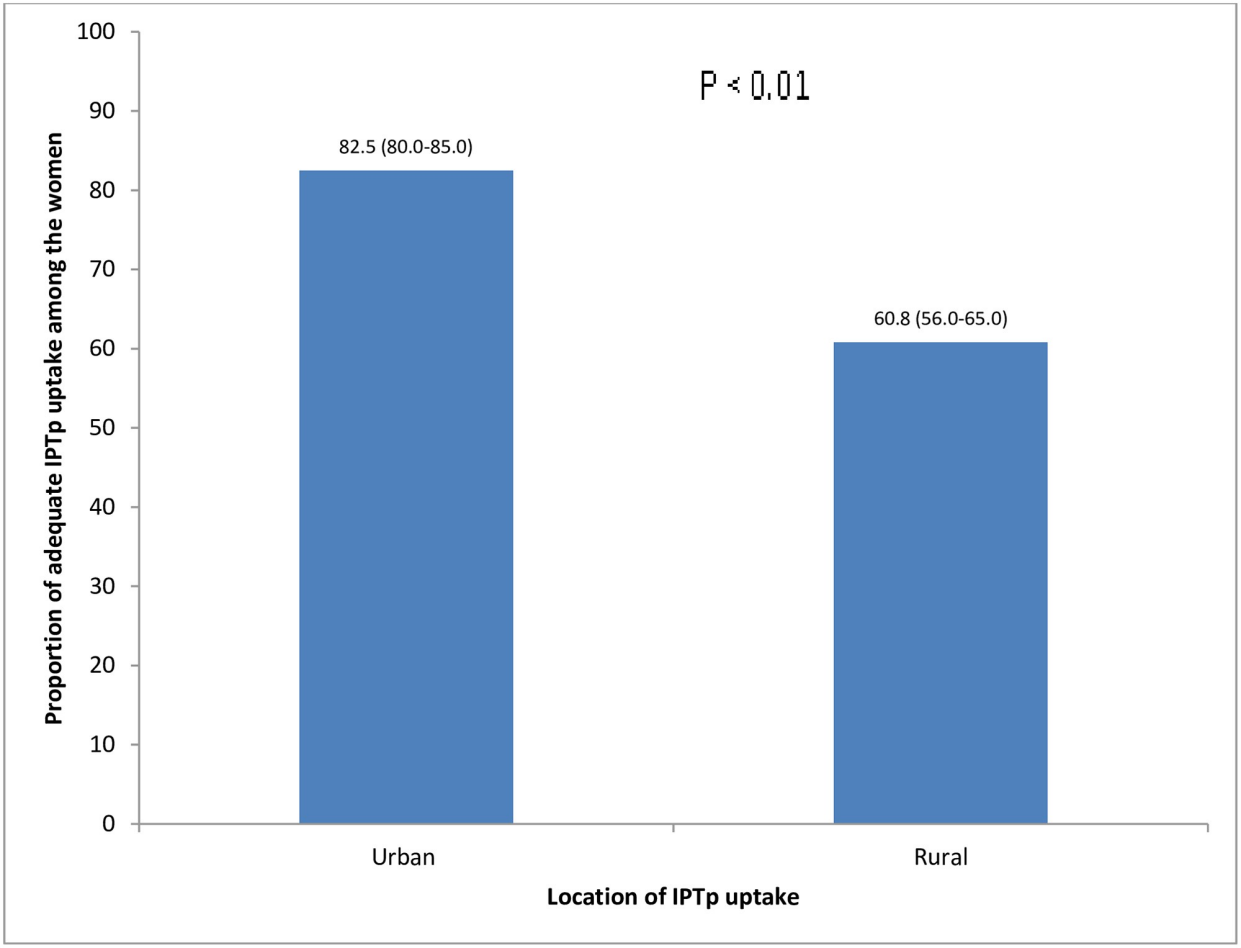


Adequate uptake of IPTp (≥3 doses) was more in the urban, 345 (82.5%) than rural area, 245 (60.8%), p <0.001 (Fig 2). The difference in the adequate uptake of IPTp in the urban and rural areas was statistically significant (Fig 2).

Respondent’s education (p = 0.040), the husband’s level of education (p = 0.015), trimester at 1^st^ ANC attendance (p = 0.036) and SP being sold in the health facility (p = <0.001) were found to be significantly associated with adequate uptake of IPTp among respondents in the urban area at bivariate analysis (Table 2). These were modeled in binary logistic regression to determine the predictors of IPTp uptake in the urban area.

**Table 2:** Association between socio-demographic characteristic, reproductive characteristics, other factors and adequate uptake of IPTp in the urban area.

Variable	Adequate uptake (%)	Inadequate uptake (%)	Prevalence of adequate uptake (%)	Unadjusted OR (95%CI)	P–value
**Total**	345 (100.0)	73 (100.0)	82.5 (78.7–86.0)		
**Age (years)**					
18–24	65 (18.9)	20 (27.4)	76.5 (66.6–84.6)	1	
25–34	243 (70.4)	40 (54.8)	85.9 (81.4–89.6)	1.87 (1.02–3.41)	**0.042** *
≥35	37 (10.7)	13 (17.8)	74.0 (60.6–84.7)	0.88 (0.39–1.96)	0.747
**Marital status**					
Married	344 (99.7)	72 (98.6)	82.7 (78.8–86.1)	1	
Unmarried	1 (0.3)	1 (1.4)	50.0 (2.5–97.5)	0.21 (0.01–3.33)	0.224
**Employment**					
Employed	128 (37.1)	35 (47.9)	78.5 (71.7–84.3)	1	
Unemployed	217 (62.9)	38 (52.1)	85.1 (80.3–89.1)	1.56 (0.94–2.56)	0.084
**Education**					
≤Primary	116 (33.6)	15 (20.5)	88.5 (82.2–93.2)	1	
≥Secondary	229 (66.4)	58 (79.5)	79.8 (74.9–84.1)	0.50 (0.25–0.97)	**0.029** *
**Ist ANC**					
1^st^ Trimester	135 (39.1)	26 (35.6)	83.9 (77.6–88.9)	1	
2^nd^ Trimester	195 (56.5)	39 (53.4)	83.3 (78.1–87.7)	0.96 (0.56–1.66)	0.892
3^rd^ Trimester	15 (4.4)	8 (11.0)	65.2 (44.5–82.4)	0.36 (0.14–0.94)	**0.036** *
**ANC attended**					
<4 time	66 (19.1)	20 (27.4)	76.7 (67.0–84.8)	1	
≥4 times	279 (80.9)	53 (72.6)	84.0 (79.8–87.7)	1.59 (0.89–2.86)	0.112
**Spouse education**					
≤Primary	23 (6.7)	11 (15.3)	67.6 (50.7–81.7)	1	
≥Secondary	321 (93.3)	61 (84.7)	84.0 (80.1–87.5)	2.52 (1.16–5.56)	**0.015** *
**SP being sold**					
Yes	258 (74.8)	69 (94.5)	78.9 (74.2–83.1)	1	
No	87 (25.2)	4 (5.5)	95.6 (89.7–98.6)	5.00 (2.08–16.67)	**<0.001** *

ANC = antenatal care, SP = Sulfadoxine-pyrimethamine, OR = odds ratio,

* = statistically significant

Respondent’s employment status (p = 0.022), trimester at 1^st^ ANC attendance (0.001) and number of ANC attended during pregnancy (p<0.001) were found to be significantly associated with uptake of IPTp among respondents in the rural area at bivariate analysis (Table 3). These were modeled in binary logistic regression to determine the predictors of IPTp uptake in the urban area. More so, husband’s employment met up with the cut-off of p = 0.1 to be modeled in binary logistic regression to determine the predictors of IPTp uptake in the rural area.

**Table 3:** Association between socio-demographic characteristic, reproductive characteristics, other factors and adequate uptake of IPTp in the rural area.

Variable	Adequate uptake (%)	Inadequate uptake (%)	Prevalence of adequate uptake (%)	Unadjusted OR (95%CI)	P–value
**Total**	245 (100.0)	158 (100.0)	60.8 (56.0–60.8)		
**Age (years)**					
18–24	71 (29.0)	47 (29.7)	60.2 (51.1–68.7)	1	
25–34	149 (60.8)	90 (60.0)	62.3 (56.1–68.3)	1.10 (0.69–1.72)	0.691
≥35	25 (10.2)	21 (13.3)	54.3 (39.9–68.3)	0.79 (0.40–1.57)	0.497
**Marital status**					
Married	234 (95.5)	147 (93.0)	61.4 (56.5–66.2)	1	
Unmarried	11 (4.5)	11 (7.0)	50.0 (29.8–70.2)	0.63 (0.27–1.49)	0.286
**Employment**					
Employed	138 (56.3)	107 (67.7)	56.3 (50.1–62.5)	1	
Unemployed	107 (43.7)	51 (32.3)	67.7 (60.1–74.7)	1.64 (1.08–2.50)	**0.022** *
**Education**					
≤Primary	176 (71.8)	85 (53.8)	67.4 (61.6–72.9)	1	
≥Secondary	69 (28.2)	73 (46.2)	48.6 (40.4–56.8)	0.65 (0.26–1.59)	0.330
**Ist ANC**					
1^st^ Trimester	111 (45.3)	39 (24.7)	74.0 (66.5–80.5)	1	
2^nd^ Trimester	126 (51.4)	94 (59.5)	57.3 (50.7–63.7)	0.47 (0.30–0.74)	**0.001** *
3^rd^ Trimester	8 (3.3)	25 (15.8)	24.2 (11.9–40.9)	0.11 (0.04–0.27)	**<0.001** *
**ANC attended**					
<4 times	55 (22.4)	74 (46.8)	42.6 (34.3–51.3)	1	
≥4 times	190 (77.6)	84 (53.2)	69.3 (63.7–74.6)	3.03 (1.96–4.76)	**<0.001** *
**Husband education**					
≤Primary	20 (8.5)	15 (10.1)	57.1 (40.5–72.7)	1	
≥Secondary	215 (91.5)	133 (89.9)	61.8 (56.6–66.8)	1.22 (0.60–2.44)	0.591
**SP being sold**					
Yes	196 (80.0)	128 (81.0)	60.5 (55.1–65.7)	1	
No	49 (20.0)	30 (19.0)	62.0 (51.0–72.2)	1.11 (0.64–1.75)	0.803

ANC = antenatal care, SP = Sulfadoxine-pyrimethamine, OR = odds ratio,

* = statistically significant

Husband’s level of education, respondent’s education and SP being sold in the health facilities were found to be the predictors of adequate IPTp uptake in the urban area. Respondents whose husbands had attained secondary education or more were more 2.9 times more likely to take adequate IPTp during pregnancy (aOR:2.9; 95%CI: 1.16–7.36; p = 0.023) compared to respondents whose husbands had attained primary education or less (Table 4). Respondents who had attained secondary education or more were 3.3 times less likely to take adequate IPTp during pregnancy in the urban area (aOR:0.3; 95%CI: 0.13–0.64; p = 0.002) compared to respondents who had attained primary education or less (Table 4). Respondents whose SP was sold to were 5 times less likely to receive adequate dose of SP (aOR:0.2; 95%CI: 0.08–0.64; p = 0.005) compared to respondents whose SP was not sold to during pregnancy (Table 4).

**Table 4:** Predictors of adequate IPTp uptake in the urban and rural area.

Variables	Urban area			Rural area		
	Unadjusted OR (95%CI)	Adjusted OR (95%CI)	p-value	Unadjusted OR (95%CI)	Adjusted OR (95%CI)	p-value
**Age in groups**						
< 25–18	1	1				
25–34	1.87 (1.02–3.41)	1.11 (0.35–3.56)	0.862			
≥ 35	0.75 (0.39–1.96)	0.41 (0.11–1.56)	0.191			
**Employment Status**						
Unemployed	1	1		1	1	
Employed	0.59 (0.39–1.06)	0.86 (0.38–1.96)	0.718	0.56 (0.40–0.93)	1.28 (0.78–2.30)	0.291
**Education Level**						
Primary or less	1	1				
Secondary or more	0.51 (0.25–0.97)	0.30 (0.12–0.63)	*0.002			
**Trimester at 1**^**st**^ **ANC attendance**						
1^st^ trimester	1	1		1	1	
2^nd^ trimester	0.89 (0.56–1.66)	0.67 (0.35–1.58)	0.439	0.45 (0.30–0.74)	**0.49 (0.32–0.86)**	*0.010
3^rd^ trimester	0.36 (0.14–0.94)	0.25 (0.06–1.01)	0.052	0.09 (0.04–0.27)	**0.20 (0.07–0.46)**	*<0.001
**Number of ANC attended**						
≥ 4	1	1		1	1	
< 4	0.59 (0.35–1.12)	0.55 (0.22–1.72)	0.357	0.25 (0.21–0.51)	**0.39 (0.26–0.70)**	*<0.001
**Husband completed education level**						
Primary or less	1	1				
Secondary or more	2.78 (1.16–5.56)	2.87 (1.16–7.36)	*0.023			
**SP being sold**						
Yes		1				
No	5.00 (2.08–16.67)	4.17 (1.56–12.50)	*0.005			

ANC = antenatal care, SP = Sulfadoxine-pyrimethamine, OR = odds ratio, CI = confidence interval

The trimester at 1^st^ ANC attendance and number of ANC attended during pregnancy were found to be predictors of adequate IPTp uptake in the rural area. Respondents who initiated their 1^st^ ANC attendance at 2^nd^ and 3^rd^ trimester were 2 times and 5 times < likely to take adequate IPTp (aOR:0.5; 95%CI:0.32–0.86; p = 0.01) and (aOR:0.2; 95%CI:0.07–0.46; p = <0.001) respectively compared to women who initiated their 1^st^ ANC attendance at 1^st^ trimester (Table 4). More so, women who attended ANC less than 4 times were 2.5 times less likely to take adequate IPTp uptake (aOR:0.4; 95%CI: 0.26–0.70; <0.001) compared to women that attended ANC four times and above (Table 4).

## Discussions

This study compared uptake of intermittent preventive treatment of malaria among reproductive age women who had given birth in the past year prior to this survey and are attending immunization centres with their children in rural versus urban areas of Ebonyi State, Nigeria.

A relatively more adequate uptake, ≥3 doses of IPTp was found in the urban area compared to the rural area of Ebonyi State. This may be attributed to more growing awareness on IPTp in the urban area of Ebonyi State than the rural area. It is also likely that urban dwellers have a better health seeking behavior such as early registration for antenatal care, good attendance for antenatal care services and acceptance of malaria preventive measure during pregnancy through IPTp. It could be possible that the pregnant women in the urban area of Ebonyi State have a better socio-economic and spousal support than their rural counterpart which may have contributed to the urban dwellers having a better attitude towards their health and that of their fetus which may have led to a better uptake of health services during pregnancy including IPTp. More so, it is likely that the urban health facilities have a steadier supply of SP used for IPTp from the State ministry of Health than the rural health facilities. It is also possible that support from the international agencies, partners and non-governmental organizations (NGOs) towards prevention of malaria during pregnancy go more to the urban areas of Ebonyi State than the rural areas due to the reason that the urban areas may be more accessible to them thereby improving the maternal health care services including adequate IPTp uptake in the urban areas.

The finding in this study is similar to that reported in Enugu in 2012 among 1307 respondents which reported a higher IPTp uptake in the urban areas [8]. It is possible that the health system of Ebonyi State have similarities with that of Enugu State in terms of supply of SP to the health facilities for IPTp implementation which may have led to the similarity in finding of both studies. More so, Ebonyi State shares the same location with Enugu State (Southeast Nigeria) and may also have some similarities with Enugu State in terms of the health seeking behavior of pregnant women in both places. This may have contributed to the similar findings in both studies. In addition, both studies were cross-sectional and comparative in design with a relatively large sample size; this may have contributed to the similarities in findings.

The rural urban difference was also reported in Nigeria Malaria Indicator Survey in 2015 among 8,028 which revealed that women in the urban area were more likely to receive three or more doses of IPTp than their rural counterparts [16]. It is worthy of note that the findings in this study are in keeping with that revealed in Nigeria Demographic and Health Survey, 2018 among 41,121 women of reproductive age which reported more adequate uptake among urban dwellers than rural [9]. The similarities between the finding in this study and the nation-wide surveys conducted in 2015 and 2018 may be explained by the fact that the health system across the States in Nigeria including Ebonyi may share similar characteristics in terms of provision of maternal health services of which IPTp through ANC is an integral part of.

A survey that was done in 8 sub-Saharan African countries among 18,603 respondents reported a low adequate uptake of IPTp at 29.5% [4]. The level of uptake in both the urban and rural areas in this study implies that the uptake of IPTp is improving and that pregnant women in Ebonyi State may likely have a better health seeking behavior in pursuit of prevention of malaria during pregnancy with the use of IPTp. It is worthy of note that Ebonyi State is one of the states that benefit from Presidential Malaria Initiative in Nigeria. The high prevalence revealed in our study could be due to the effect of the programme activities on malaria prevention in the State. Despite the fact that the proportion of women that had adequate uptake of IPTp (≥3 doses) in the urban and rural areas in this study was high with the urban higher than the rural, the WHO recommended target of 100% is yet to be achieved in Ebonyi State. The proportion of pregnant women who had adequate dose was higher than that reported in a cross-sectional study in an urban area of Ghana in 2019 among 382 respondents [5].

In the urban area, age, the woman’s level of education, husband’s level of education, trimester at 1^st^ ANC attendance and SP being sold at the health facilities were significantly associated with adequate uptake of IPTp. However, the woman’s level of education, husband’s level of education and SP being sold in the health facilities were found to be predictors of adequate IPTp uptake in this study. The significant association of the trimester at ANC attendance with adequate uptake is similar to the finding in a cross-sectional study conducted in 2019 in urban area of Ghana among 382 respondents [5]. This similarity may be due to the fact that both studies were conducted in 2019 and that similar trends in terms of factors associated with adequate uptake of IPTp may be found in sub-Saharan African countries especially among studies conducted the same period. This is because the sub-Saharan African countries share a lot of socio-economic and cultural factors in common that could interact with the health seeking behavior of pregnant women especially towards ANC services. The findings from our study suggests that increased awareness on the importance of IPTp may be needed among the women with high level of education in the urban since our study revealed that women with high level of education in the urban were less likely to have taken adequate dose of IPTp. Nevertheless, Nigeria malaria indicator survey 2015 reported that woman’s education was a predictor of adequate IPTp uptake in the positive direction as more educated women were more likely to have taken adequate dose [16].

In contrast, our study equally revealed that male education promotes adequate IPTp uptake among pregnant women in the urban. It is possible that educated husbands in the urban are more likely to be involved in antenatal care services and provide support to their spouses such as encouraging them to take IPTp as advised by the health workers to prevent malaria in pregnancy. There is a likelihood they may have better risk perception and encourage their spouses to adhere to recommendations. It is worthy of note that studies in Nigeria as well as Uganda have reported lack of Knowledge on the relevance of IPTp formed a huge barrier to its uptake [17, 18]. More so, payment for SP used for IPTp constituted a barrier to adequate IPTp uptake in the urban. There exists a policy in Ebonyi State that SP used for IPTp should be given free but it appears from our findings that this policy is not completely being implemented in all Health facilities in the urban.

In the rural area, woman’s level of education, woman’s employment status, trimester at 1^st^ ANC attendance and number of ANC attended before delivery were associated with adequate IPTp uptake but only the trimester at 1^st^ ANC attendance and number of ANC attended before delivery remained predictors of adequate IPTp uptake in this study. There was a similarity in the factors significantly associated with adequate uptake and predictors of adequate uptake of IPTp in this study with a cross-sectional study that was conducted in 2019 in urban Ghana among 382 respondents [5]. Also, a cross-sectional study in 2012 in Ghana among 253 respondents equally reported the number of ANC attendance during pregnancy as a predictor of adequate uptake of IPTp [19]. This is in keeping with the finding in our study. It is worthy of note that pregnant women who initiate ANC attendance as early 1^st^ trimester have more opportunities to attend more ANC before delivery, have more contacts with the healthcare workers hence, have more tendencies/opportunities to take adequate IPTp (≥3 doses) before delivery. Our findings reveal the fact that there may be a good awareness campaigns on the importance of early initiation of ANC attendance and adherence to ANC appointments in the rural areas of Ebonyi State, Nigeria.

### Limitations

A limitation of this study was the possibility of social desirability bias. To minimize this, the questionnaire was administered to the respondents in a non-judgmental manner so as to not influence their responses. In addition, there was a possibility of recall bias. However, to ameliorate this, the respondents were shown pictures of Sulfadoxine/Pyrimethamine package to help them identify the medicine and correctly recall taking it. More so, the study included only women who had given birth in the past year prior to the survey to help their recall. The hospitals do not routinely keep adequate records of IPTp administration and mode of intake especially in the rural areas making necessary to interview the patients.

## Conclusions

Adequate IPTp uptake was significantly more in the urban than rural areas of Ebonyi State. Whereas the predictors of adequate IPTp in the urban were husband’s education, woman’s education and payment for IPTp, the predictors in the rural area remained the trimester at 1^st^ ANC attendance and the number of ANC attended during pregnancy.

Interventions aimed at reinforcing the importance of health professionals to carry out actions aimed at pregnant women and their partners (spousal) in order to guide them on preventive actions against malaria and other diseases are recommended in Ebonyi State.

## Supporting information

S1 Questionnaire Study questionnaire. (DOCX)

## Abbreviations

ANC: Antenatal care
DOT: Directly observed therapy
IPTp: Intermittent preventive treatment in pregnancy
ITN: Insecticide treated nets
LGAs: Local government areas
NGOs: Non-governmental organizations
REC: Research and ethics committee
SP: Sulfadoxine-pyrimethamine
WHO: World Health Organization
