# Supplementary material for: Are rural pregnant women disadvantaged in accessing intermittent preventive treatment in pregnancy in Ebonyi State, Nigeria?
Source: PLoS One. 2022 Nov 10;17(11):e0269305. doi: 10.1371/journal.pone.0269305 (PMC9648760; doi:10.1371/journal.pone.0269305)
Supplement: S1 Questionnaire — (DOCX) [file pone.0269305.s001.docx]

Study questionnaire

**QUESTIONNAIRE**

**RESEARCH QUESTIONNAIRE ON INTERMITTENT PREVENTIVE TREATMENT IN PREGNANCY UPTAKE AND UTILIZATION: AN URBAN-RURAL COMPARATIVE STUDY AMONG REPRODUCTIVE AGE WOMEN ATTENDING IMMUNIZATION CENTRES IN EBONYI STATE, NIGERIA.**

*Dear Respondent,*

*I am carrying out a study on the above subject matter. Any information you will provide will be treated with absolute confidentiality and will neither be disclosed to other persons nor be used against you in any way.*

Code No……………………..

**SECTION A: SOCIODEMOGRAPHIC AND HOUSEHOLD CHARACTERISTIC**

1. Age in years………………. Years
2. Marital status:1. Married [ ]2. Single [ ] 3. Divorced [ ] 4. Separated [ ]
3. Employment status:1. Employment[ ] 2.Unemployed [ ]
4. Occupation: ………………………………………………………..
5. Religion: 1. Protestant [ ] 2. Pentecostal [ ] 3. Catholic [ ] 4. Islam [ ] 5. Others [ ]Please specify……………………………………………………
6. Level of education completed: 1. None[ ] 2. Primary [ ] 3. Secondary [ ] 4.Tertiary [ ]
7. How many times have you had live births? 1. 1 [ ] 2. 2-4 [ ] 3. > 4 [ ]
8. What is the age of your last child? (in completed weeks/months)……………….
9. What is your husband’s completed level of education?1. None [ ] 2. Primary [ ] 3. Secondary [ ] 4. Tertiary [ ]
10. What is your husband’s employment status? 1. Employed [ ] 2.Unemployment [ ]
11. What is your husband’s occupation? ……………………………….
12. Who is the head of your household: 1. Husband/Partner [ ] 2. Myself [ ] 3. Father-in-law [ ] 4. Mother-in-law [ ] 5.Husband/Partner’s elder brother 6. Other please specify……………..
13. How many are you in your household……………….
14. Type of family: 1. Monogamous [ ] 2. Polygamous [ ] 3. Others please specify………………………………

15. Trimester of 1^st^ ANC attendance during last pregnancy: 1. 1^st^ Trimester [ ] 2. 2^nd^

2^nd^ Trimester [ ] 3. 3^rd^ Trimester [ ]

16. Number of ANC attended during last pregnancy: 1. Once [ ] 2. Twice 3. Three times [ ]

4. Four times and above [ ]

**SECTION B: UPTAKE OF IPTp**

17. Did you take any anti malaria medicine during your last pregnancy? 1. Yes [ ] 2. No [ ]

1. If yes, how many the tablets? 1. 1 [ ]2. 2 [ ] 3. 3[ ] 4. 4 [ ]
2. If ‘3’, how many times did you take it before you gave birth? 1. Once [ ] 2. Twice [ ] 3. Three times [ ] 4. More than three times [ ]
3. Did you receive it during ANC visit? 1. Yes [ ] 2. No [ ]
4. If yes, what is the name of the health facility you received it…………………………… then continue with section with section C

If ‘No’, move to section D

1. Did you pay for Sufadoxine pyrimethamine given to you for IPTp in the Health facility?
2. Yes [ ] 2. No [ ]

**SECTION C: Directly observed therapy (DOT) in the administration of IPTp**

1. When was this given to you in the clinic? 1. After seeing the Health worker 1.Yes [ ] 2. No [ ]
2. Who gave it to you? 1. Doctor [ ] 2. Nurse [ ] 3. CHEW [ ] 4 Pharmacist/Drug Dispenser [ ]

1. I took it in the health facility under the observation of the health worker? 1. Yes [ ]2. No [ ]
2. Did the health worker counsel you on taking SP by DOT? 1 Yes [ ] 2. No [ ]
